# New Appliances and Things Medical

**Published:** 1906-09-15

**Authors:** 


### NEW APPLIANCES AND THINGS MEDICAL.

[We shall be glad to receive at our Office, 28 & 29 Southampton Street, Strand, London, W.C., from the manufacturers, specimens of all new preparations and appliances which may be brought out from time to time.]

### CLAXTON'S CRUTCH BED-REST.

(A. CLAXTON, 62 STRAND, LONDON, W.C.)

WE have received a specimen of this most ingenious bed-rest crutch. It consists essentially of a pair of exceedingly well-padded crutches, which can be applied to any ordinary bed-rest, and which fit under the patient's arms, and thus maintain him in the sitting posture. All those who have experience in the nursing of patients suffering from orthopnœa know full well what a tax is placed upon the nurse by the continual raising of a patient, which, in spite of pillow arrangement, however ingenious, is constantly necessary with the ordinary bed-rest. The use of these crutches, which can be applied at any height on the bed-rest, greatly diminishes the patient's tendency to slip down. Our experience with them has shown us that they are of the greatest possible assistance to nurses and patients alike, in that being raised is as trying to the patient as raising is to the nurse. We would further point out that the exceptionally careful way in which the crutches are padded, and the fact that some lateral movement between the crutch and the bed-rest is allowed by the method of application of the former to the latter, prevents any pressure being exerted by the crutches upon the structures in the axilla. We think this apparatus is exceedingly ingenious, and in every way to be recommended.

### EKENBERG POWDERED MILK.

(MANUFACTURED IN SWEDEN BY THE EKENBERG MILK PRODUCTS COMPANY, LIMITED, LONDON. LONDON AGENTS : MESSRS. TRAVERS AND SONS, LIMITED, 119 CANNON STREET, E.C.)

THE convenience of dry milk or milk flour is now established beyond dispute, and this is true whether we wish for milk as a beverage or simply to incorporate milk for mixing with other flours. The milk flour before us, for instance, is eminently suited for making biscuits, cakes, pastry, or shortbread; and in that it is entirely free from any lactic acid bacteria, the rising of dough takes place more completely under its influence than under that of fresh milk. Further, the incorporation of milk flour with ordinary flour for biscuit and bread making greatly increases the nutritive value of the finished product. We have received three varieties of Ekenberg powdered milk. These differ essentially in the amount of fat which they contain. The first quality contains approximately 20 per cent. of fat, and when made up according to the directions into liquid milk the finished product contains approximately as much fat as average milk. The other two qualities contain very much less fat and the milk made from them corresponds roughly to hand-skimmed and separated milk respectively. In all cases, however, the milk made from these powders has a good taste and pleasant appearance, in fact may be regarded as milk.

### COCOIDS OF DIASTASE.

(OPPENHEIMER, SON AND COMPANY, LIMITED, 179 QUEEN VICTORIA STREET, LONDON, E.C.)

WE have repeatedly in these columns drawn attention to the extreme value of diastase in certain forms of amylaceous dyspepsia, especially in children. In a series of notices which we wrote upon malt extracts we pointed out that the administration of the active ferment played often quite as large a part in the beneficial effects which arose from their use as the actually nutrient part of the preparation itself. Messrs. Oppenheimer, Son and Company, who are well known to the profession as assiduous and successful workers in this field of research, have succeeded in developing a new diastase from malted barley of very high activity. They have put up this ferment into two exceedingly convenient forms—namely, cocoids and platinoids, each containing 5 grains. We have made a trial of the cocoids which have been submitted to us, and find them in every way satisfactory.

